# Current status and perspectives of patient-derived xenograft models in cancer research

**DOI:** 10.1186/s13045-017-0470-7

**Published:** 2017-05-12

**Authors:** Yunxin Lai, Xinru Wei, Shouheng Lin, Le Qin, Lin Cheng, Peng Li

**Affiliations:** 1 Key Laboratory of Regenerative Biology, South China Institute for Stem Cell Biology and Regenerative Medicine, Guangzhou Institutes of Biomedicine and Health, Chinese Academy of Sciences, Guangzhou, 510530 China; 2 Guangdong Provincial Key Laboratory of Stem Cell and Regenerative Medicine, South China Institute for Stem Cell Biology and Regenerative Medicine, Guangzhou Institutes of Biomedicine and Health, Chinese Academy of Sciences, Guangzhou, 510530 China; 3 Department of Abdominal Surgery, Affiliated Cancer Hospital & Institute of Guangzhou Medical University of Guangzhou Medical University, Guangzhou Medical University, Guangzhou, Guangdong 510095 China

**Keywords:** PDX models, Basic, Preclinical, Cancer research, Drugs

## Abstract

Cancers remain a major public health problem worldwide, which still require profound research in both the basic and preclinical fields. Patient-derived xenograft (PDX) models are created when cancerous cells or tissues from patients’ primary tumors are implanted into immunodeficient mice to simulate human tumor biology in vivo, which have been extensively used in cancer research. The routes of implantation appeared to affect the outcome of PDX research, and there has been increasing applications of patient-derived orthotopic xenograft (PDOX) models. In this review, we firstly summarize the methodology to establish PDX models and then go over recent application and function of PDX models in basic cancer research on the areas of cancer characterization, initiation, proliferation, metastasis, and tumor microenvironment and in preclinical explorations of anti-cancer targets, drugs, and therapeutic strategies and finally give our perspectives on the future prospects of PDX models.

## Background

Cancers are among the leading causes of death worldwide. The Cancer Moonshot 2020 program has been launched in 2016 to transform the cancer research and care ecosystem and double the rate of progress in cancer prevention, diagnosis, and treatment [1], though success achieved in reducing cancer death rates in the USA [2]. This program envisaged the development of precision medicine based on five critical elements—clinical bioinformatics, precision methods, disease-specific biomarkers, drug discovery and development, and precision regulations—to guard the application of precision medicine [3]. Novel techniques and research tools would play important roles in this process.

**Fig. 1:**
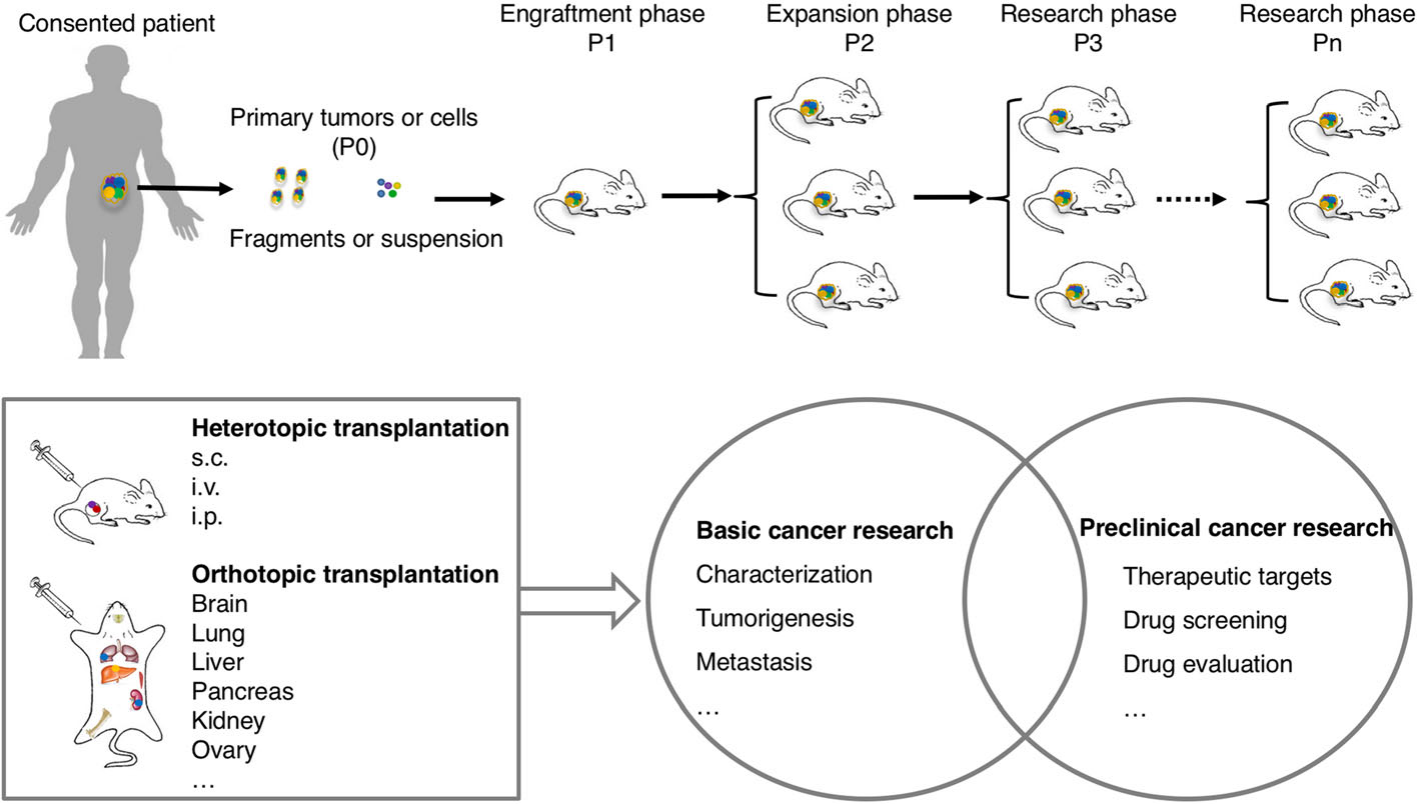
Overview of the methodology to establish PDX models and their uses in cancer research. Tumors from cancer patients (*P0*) are fragmented or digested into single-cell suspension and then transplanted (directly or with additives such as Matrigel) into immunodeficient mice (*P1*) for engraftment. Once grown, the tumors were transplanted into secondary recipients (*P2*) for tumor expansion. The expanded tumors can then be cryopreserved or transplanted into *P3* mice for cancer research of the type of origin. Specifically, tumors can be transplanted into the sites other than that the tumors are derived, called heterotopic transplantation or into the corresponding sites of the tumors like the brain [39, 97], lung [130], liver [12], pancreas [131, 132], kidney [26],and ovary [11], which is called orthotopic transplantation. The successfully established PDX models are to be used in cancer research, which consists of two, basic and preclinical, arms. Basic and preclinical cancer research in PDX models are connected with each other, as basic research can identify therapeutic targets or strategies for preclinical tests and preclinical research can generate new basic questions

**Fig. 2:**
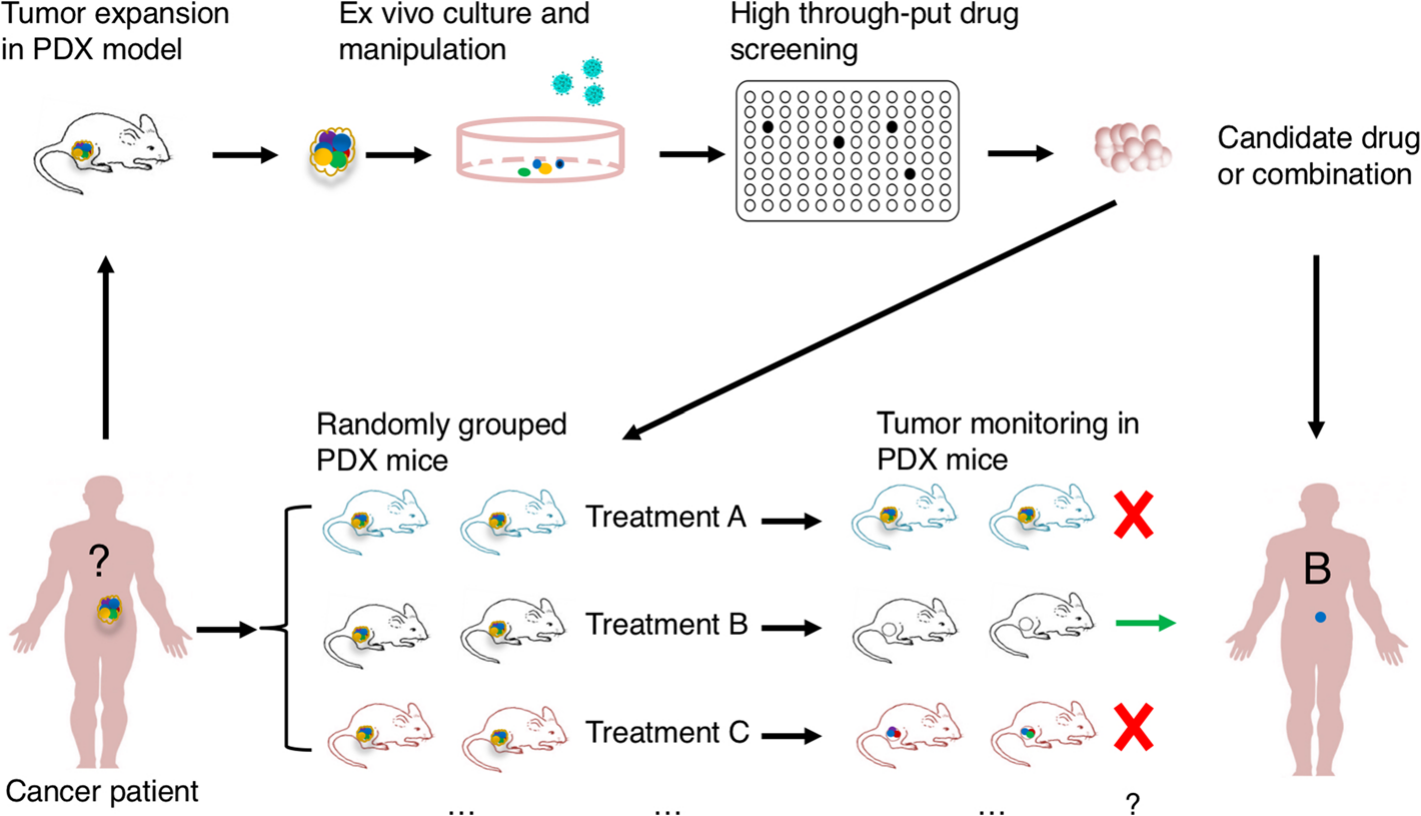
Use of PDX models in drug screening and preclinical therapeutic evaluation. Drug screening: PDX models can be used to expand tumors derived patients without adequate initial tumors for in vitro studies. The expanded tumor cells can be cultured and manipulated ex vivo and used for high-throughput screening of drugs or combinations. Identified candidate drugs and combinations can be further evaluated in PDX mice before use in patients or directly used in patients if the drugs have been approved. Preclinical therapeutic evaluation: given different clinical therapeutic regimens are available for cancer patients, PDX models can be used to define the best for individual patients. Briefly, the PDX mice of one patient are randomly divided into certain groups and treated with different therapeutic regimens. Through tumor assessment, the best regimen can be identified

Patient-derived xenograft (PDX) models are immunodeficient mice engrafted with patients’ cancerous cells or tissues. The development of PDX models for cancer research, based on the assumption that these models faithfully resemble the original tumors, especially for the patient-derived orthotopic xenograft (PDOX) models [4], has significantly enhanced cancer research in recent years. These models for various types of cancers, such as chronic lymphocytic leukemia [5], large B cell lymphoma [6], pancreatic cancer [7], colorectal cancer [7, 8], gastric cancer [9, 10], high-grade serous carcinoma [11], and intrahepatic cholangiocarcinoma [12], are biologically stable and accurately reflect the patients’ tumors with regard to histopathology, gene expression, genetic mutations, inflammation [13], and therapeutic response. Thus, PDX models allow invaluable assessment of human tumor biology, identification of therapeutic targets, and preclinical screening and evaluation of drugs for various cancers. In this review, we summarize the methodology to establish PDX models (Fig. 1), go over the recent advances of basic cancer studies and preclinical studies in which PDX models have been used (Fig. 2), and give our perspectives on the future prospects of PDX models.

## Methodology to establish PDX models

### Immunodeficient mice

Immunodeficient mice engrafted with human immune systems provide powerful models for the study of human immunobiology in vivo, and PDX models with these humanized mice are critical tools for studying the interaction between human immunity and various cancers. In order to establish a PDX model, we need a highly immunodeficient mouse strain. Several types of immunodeficient mice can be used to establish xenograft models: athymic nude mice, SCID, NOD-SCID, and recombination-activating gene 2 (Rag2)-knockout mice [14]. However, these strains are usually used to establish cancer cell line xenograft models. Primary cancerous cells or tissues require higher immunodeficiency for efficient engraftment in mice. NOD/SCID mice with IL2rg mutations, such as NOD.Cg-*Prkdc* ^*scid*^ *Il2rg* ^*tm1Wjl*^ (NSG) [15] and NODShi.Cg-*Prkdc* ^scid^ *Il2rg* ^tm1Sug^ (NOG) mice [16], are with enhanced immunodeficiency and able to engraft almost all types of human cancers [17–20]. We generated a strain of NOD/SCID/IL2rg^−/−^ (NSI) mice, which exhibit severe immunodeficiency, lacking T, B, and NK cells, and used these mice in studies of both leukemia and solid tumors [21–25]. As the number of immunodeficient strains increases, the choice of mouse strains for cancer research matters. We developed a method to quantitatively evaluate the immunodeficiency of various strains of mice, through the tumor engraftment index (TEI) [21]. Recently, we also derived a nude train of NOD/SCID/IL2rg^−/−^ mice, called NSIN, by deleting *foxn1* with CRISPR/Cas9 system. The nude NSIN mice showed even higher immunodeficiency than NSI mice by TEI and can be more suitable for studies of tumors with poor engraftment efficiency (data unpublished).

### Primary tumor samples

For the first implantation, patient-derived tumors may be implanted into immunodeficient mice in the form of small tumor fragments or cell suspensions derived from blood of patients or from digestion of tumors into single-cell suspensions. Principal determinants of successful tumor engraftment into immunodeficient mice are the viability and sterility of the human tumor [26]. Cancer cells or tissues can be mixed with basement membrane matrix proteins (Matrigel) before injected into recipient animals, which enables the growth of tumors with greater efficiency of take and growth [27], without loss of the primary tumor phenotype [28]. Tumor cells can also be co-injected with additional cell types, such as fibroblasts, stromal cells, and endothelial cells, according to experimental objectives.

### Heterotopic vs orthotopic implantation

Cancerous cells or tissues can be implanted heterotopically or orthotopically and monitored for tumor formation (Fig. 1). In contrast to orthotopic implantation, heterotopic implantation has advantages including easy methods of cell implantation, accurate monitoring of tumor size. Subcutaneous and intravenous PDX models, for solid tumors and leukemia, respectively, are most widely used in cancer research. Orthotopic implantation is more technically challenging and time-consuming and often requires ultrasound examinations or exploratory laparotomies to confirm the presence of tumors inside; however, the advantage is that the external milieu is more closely preserved in orthotopic tumors and theoretically better approximates the “natural” setting of human tumors. Orthotopic implantation can increase the incidence of metastases during xenograft growth and should be considered when tumor metastasis is the investigation subject [29]. To improve the engraftment efficiencies of inadequate quantities of patient-derived tumors, it is favorable to do the initial subcutaneous implantation of patient-derived tumors into F1 mice. Once grown, the tumor may then be digested and orthotopically transplanted into subsequent generations of mice.

### Induced pluripotent stem cells (iPSC)-derived PDX models

Since many patients’ primary tumors cannot engraft directly in immunodeficient mice, other methods are needed to establish PDX models for these patients. Primary tumor cells can be reprogrammed to iPSC and then differentiated into the cell type of origin, which then can be used to establish PDX models. PDX models derived through an intermediate iPSC stage could be useful in approximately one third of patients whose primary cells cannot undergo PDXs [30]. An advantage of this method is that an intermediate iPSC stage enables the genetic manipulation of the cells in vitro before transplantation to facilitate tracking or study of their effects on tumor growth in vivo.

### Next-generation PDX models with humanized mice

Recent advances in immunotherapies highlight the importance of the immune system in tumor progression and treatment, which require PDX models with human immune system to facilitate the study of immunity-cancer interactions and preclinical assessment of cancer immune therapies. To establish human immune system-conditioned PDX models, we first need to generate humanized mice (also known as human hemato-lymphoid chimeric mice or human immune system models). One method for the generation of humanized mice involves the transplantation of total peripheral blood or tumor-infiltrating lymphocytes (TILs) into immunodeficient mice. These procedures are known to cause severe graft versus host disease (GVHD) 2–5 weeks after injection [31] and limit the useful investigative time window [32]. Another method is to transplant CD34-positive human hematopoietic stem cells (HSCs) or precursor cells isolated from the umbilical cord blood, bone marrow, and peripheral blood, either alone or in combination with additional human immune tissues (e.g., human thymic tissue) into immunodeficient mice [33]. Transplantation with HSCs results in a more complete hematopoietic reconstitution, as HSCs give rise to various lineages of human blood cells in mice. To improve the integrity of engrafted human immune system, genetically modified immunocompromised mouse strains have been generated, such as NOG-GM3, NSG-SGM3, and MISTRG [34]. The next-generation PDX models based on genetically and immune cells humanized mice, though expensive, are to be used widely in future cancer research.

## PDX models in basic cancer research

Basic cancer research is to characterize cancer biology and explore mechanisms involved for improved understanding or prediction of cancer. PDX models essentially provide important in vivo and ex vivo evidence to aid basic studies of cancer, including tumor characterization, tumorigenesis, and metastasis.

### Characterization of cancer biology

Provided that PDX models faithfully mimic human cancers, they can be used to delineate the per se molecular, cellular, and sub-clonal characterizations of various types of cancers. In the PDX model of acute lymphoblastic leukemia (ALL), a rare unfavorable ALL subpopulation has been defined which is dormant and treatment resistant and mimics patients’ primary cells at minimal residual disease [35]. PDX models of acute myeloid leukemia (AML) were used to study the relationships between clonal architecture and functional heterogeneity, in which subclones showed variable engraftment potential in immunodeficient mice and xenografts were predominantly comprised of a single genetically defined subclone [36]. For solid tumors, intratumoral heterogeneity arises from the evolution of genetically diverse subclones during tumor progression, and PDX models are ideal tools for studying the stability, the proliferation, persistence, chemotherapy tolerance, and the mechanisms involved [37]. PDX models revealed that tumor growth can be driven by a minor cell subpopulation, which enhances the proliferation of all cells within a tumor by overcoming environmental constraints and yet can be outcompeted by faster proliferating competitors, resulting in tumor collapse [38].

### Tumorigenesis

PDX models are frequently used to study the cellular components involved in cancer cell initiation and proliferation. The cancer stem cell (CSC) hypothesis suggests that neoplastic clones are maintained exclusively by a rare fraction of cells with stem cell properties. Xenograft assay identified CD133^+^ human brain tumor initiating cells (TICs) that initiate tumors in vivo, providing insights into human brain tumor pathogenesis, giving strong support for the CSC hypothesis as the basis for many solid tumors [39]. The intrinsic molecular mechanisms of tumorigenesis are usually studied in cancer cell line xenograft (CCLX) models, in which cancer cell lines were genetically modified, to consolidate in vitro studies. For examples, LZAP inhibits, by the evidence from cancer cell line xenografts that decreased LZAP expression promoted, tumor growth and vascularity [40]; knockdown of endogenous PCBP1 enhanced tumorigenesis whereas overexpression of exogenous PCBP1 abrogated tumor formation [41]; Notch- and Hedgehog-dependent TICs were identified in prostate cancer CCLX models [42]; short hairpin RNA (shRNA) targeting long non-coding RNAs (lncRNAs) in castration-resistant prostate cancer cell lines strongly suppressed tumor xenograft growth in vivo [43]. Since in vitro expansion and genetic manipulation of primary tumor cells are difficult, we can use PDX models for tumor cell expansion and molecular targeting (inhibitors or agonists). Musashi (Msi) is a critical element of pancreatic cancer progression, and Msi inhibition blocked the growth of primary patient-derived tumors [44]. The initiation of human neuroendocrine prostate cancer from prostate epithelial cells is driven by N-Myc and activated AKT1, as evidenced by the in vivo transformation in NSG mice of prostate basal epithelial cells overexpressing N-Myc and myrAKT1 [45]. MiRNA-126 stabilizes B-ALL in a proliferative B cell precursor state by targeting cell cycle/apoptosis and p53 response genes and antagonizing miRNA-126 in human B-ALL reduces disease burden in its PDX model [46]. Millions of somatic mutations have been found in cancers through genome sequencing, but the functional impact of most mutations is poorly understood. With the help of PDX models, we can define the impactful mutations that induce tumor formation and/or confer resistance to therapy [47]. The proliferation of human cancer cells can be easily defined or compared through the growth of cancer cells in PDX mice. Human cancer cells in PDX models increase growth rate with time per se without treatment [48]. A method was established for identifying novel cancer targets via negative-selection RNAi screening using a human breast cancer xenograft model at an orthotopic site in the mouse, by which a set of metabolic genes associated with aggressive breast cancer and stemness were screened to identify those required for in vivo tumorigenesis [49].

### Metastasis

Metastasis is the basis of cancer lethality, of which the mechanisms are not fully understood and interventional strategies not well defined. PDX models are useful in defining cell populations and molecules associated with metastasis. Metastasis-initiating cells (MICs) have been proven critical for cancer metastasis. But it is difficult to identify and isolate adequate numbers of MICs from patients for research. PDX models are depositories of MICs. PDX model of human breast cancer was used to identify and isolate MICs through a highly sensitive fluorescence-activated cell sorting (FACS)-based assay [50]. Circulating tumor cells (CTCs) play a critical role in tumor metastasis and have been identified and isolated from patients with several tumor types. Isolated CTCs have been used to generate PDX models of breast [51], pancreatic [52], and prostate cancers [53]. And these PDX models are ideal for the study of tumorigenecity, phenotypic and genetic characterizations of CTCs [54]. Recently, both CCLX and PDX models were used to assess the effect of blocking the fatty acid receptor CD36 on the metastasis of cancer which revealed CD36 as an anti-metastasis target [55]. Elsewhere, the relationship between metastasis and P53 deficiency was studied in PDX models of triple-negative breast cancer [56].

## PDX models in preclinical cancer research

Anti-cancer therapies exert selective pressure on tumor cells that leads to the preferential growth of resistant subpopulations, necessitating the development of novel generations of therapies to treat the evolving cancers. A critical role for PDX models in preclinical research is to identify therapeutic targets, including specific molecules and molecular interactions. Another major role for PDX models is as a guide for the clinical treatment of cancer patients (Fig. 2). The choice of therapeutics is critical for cancer treatment and is dependent on the cancer type and the patient. PDX models provide solutions to the challenges that researchers face in cancer drug research such as positive tumor responses in mouse models but not translating over when the study is implemented in humans.

First, PDX models can help to discriminate the most suitable therapy for cancer patients (Fig. 2). PDX models can be used to identify patients with cancers that are resistant to chemotherapy [57] and define the association between drug resistance and genetic mutations [58]. Second, PDX models can be used to identify and evaluate new anti-cancer therapeutic approaches, including new conventional chemotherapies, surgery, radiation, and also the less common microwave, nanoparticles, genetic therapies. For examples, encapsulating BYL719, a PI3Kα inhibitor, into P-selectin-targeted nanoparticles led to specific accumulation of BYL719 in the tumor milieu of PDX model for head and neck squamous cell carcinoma [59]; transdifferentiation-induced neural stem cells which were genetically engineered with optical reporters and tumoricidal gene were evaluated effective in globlastoma PDX models [60]; precise fluorescence-guided surgery (FGS) has the potential to greatly improve outcomes for patients with recalcitrant cancers. During development, the technique was preclinically evaluated in a PDX model of pancreatic cancer, in which cancer and stroma cells were labeled with different colors [61] and a PDX model of colon cancer was also used for FGS with fluorophore-conjugated anti-CEA antibody [62]. The preclinical studies of radiation therapies in PDX models have been reviewed elsewhere [63]; a lung cancer cell line xenograft model has been used for evaluation of microwave hyperthermia therapy [64]; however, PDX models have been rarely reported in the evaluation of microwave hyperthermia therapy. Third, which is the most important, PDX models are useful for preclinical drug tests which can indicate drug safety, efficacy, and dosage. PDX models have been applied to preclinical drug testing in many different types of cancers, including pancreatic cancer [65], non-small cell lung cancer (NSCLC) [66, 67], melanoma [68], breast cancer [69, 70], colon cancer [71], and prostate cancer [72]. PDX model-based oncology drug development in specific cancers has been discussed comprehensively [73].

CCLX models are not adequate for preclinical development of anti-cancer agents because most human cancer cell lines do not accurately reflect human malignant tumors [74]. In contrast, PDX models can better recapitulate each individual patient’s cancer pathology. The use of these models for in vivo preclinical investigations would yield results more predictive of subsequent activity in patients. PDX models provide in vivo platforms to study the mechanisms by which anti-tumor agents exert their effects and the cellular and molecular mechanisms of therapy resistance of cancers [75, 76]. Here, we give a brief summary of preclinical cancer research which uses PDX models to identify and evaluate therapeutic targets, varied kinds of anti-cancer “drugs” and therapeutic approaches. Representative drugs and their targets are shown in Table 1.

**Table 1:** Representative potential therapeutic drugs and their targets in various types of cancers that have been assessed by xenograft models [133]

Drug or combination	Target	Cancer type	Mouse	References
DEL-22379	Erk	Colorectal cancer	NOD/SCID	[134]
CSL362	CD123	AML	NSG	[135]
Bicalutamide	Androgen	Prostate cancer	SCID	[72]
FP3	VEGF	Colon cancer	Nude	[71]
Pyruvinium pamoate	Glutathione	Lymphoma	NOG	[116]
Ponatinib, dovitinib, and BGJ398	FGFR	Cholangiocarcinoma	NSG	
Luteolin	cMet	Gastric cancer	Nude	[95]
BKM120	PI3K inhibitor	Pancreatic adenocarcinoma	NSG	[52]
Erlotinib and gefitinib	EGFR	Chordomas	Nude	[86]
Salmonella A1-R	-	Melanoma	Nude	[104]
Salmonella A1-R and doxorubicin	-	Sarcoma	Nude	[103]
Trastuzumab	Her2	Esophageal squamous cell carcinoma	Nude and SCID	[99]
Trastuzumab/cetuximab	Her2/EGFR	Gastric cancer	Nude	[98]
Cetuximab/bevacizumab	EGFR/VEGF	Colon cancer	Nude	[136]
Cetuximab	EGFR	Lung Adenocarcinoma	NOD/SCID	[87]
AZD5363	AKT	Gastric cancer	Nude	[88]
Brequinar	Dihydroorotate dehydrogenase	AML	SCID	[137]
GSK2879552	LSD1, lysine demethylase 1	Small cell lung cancer	Nude	[138]
Anti-CD47 antibody	CD47	Non-Hodgkin lymphoma	NSG	[114]
CHZ868	JAK2	B-ALL	NSG	[139]
HA15	Bip	Melanoma	nude	[140]
UNC0379	SETD8	Neuroblastoma	Nude	[141]
PARP inhibitors and β-lapachone	DNA repair	Pancreatic cancer and NSCLC	NOD/SCID	[142]
MCB-613	Steroid Receptor Coactivator	Breast cancer (MCF-7)	Nude	[143]
P5091	USP7	Multiple myeloma	SCID	[144]
MLN8237 and ABT-199	Aurora kinase and BCL-2	Neuroblastoma	SCID	[111]
TH287 and TH588	MTH1	Melanoma	NOG	[93]
Agonists	HIF-2	Renal cell carcinoma	Nude	[145]
SSR128129E (SSR)	FGFR	Lewis lung carcinoma And breast cancer	Nude	[146]
CH5424802	ALK	NSCLC	SCID or nude	[147]
ON01910	Plk1	Liver, breast, and pancreatic cancers	Nude	[148]
Shepherdin	ATP pocket of Hsp90	Prostate cancer	SCID and beige	[149]
PD0325901	MEK	BRAF mutant cancer	Nude	[150]
Monoclonal antibody	S1P	Multiple cancers	Nude	[151]
NSC23766	Rac	P210-BCR-ABL positive CML	NOD/SCID	[152]
Argyrin A	Proteasome	Colon cancer	Nude	[153]
Syk inhibitors	Syk	AML	NOG	[154]
Polyphenylureas	XIAP, an apoptosis suppressor	Prostate and colon cancers	Nude	[155]
RD162 and MDV3100	Androgen	Advanced prostate cancer	SCID	[156]
EPI-001	Androgen receptor NTD domain	Castrate-recurrent prostate cancer	NOD/SCID	[157]
piperlongumine	Stress response to ROS	Multiple cancers	nude	[158]
CFI-400945, inhibitor	PLK4	Multiple cancers	NSG and SCID	[159]
BDA-366	Bcl2 BH4 domain	Lung cancer	Nude	[160]
CCT196969, CCT241161	pan-RAF and SFKs	Multiple cancers	Nude	[161]
SR9243, LXR inverse agonist	LXR	Multiple cancers	Nude	[162]
SHP099	SHP2	RTK-driven cancer	Nude	[163]
Antibody	RSPO3	Colorectal cancer	Nude	[164]
CB-5083	AAA ATPase p97	Multiple myeloma and solid tumors	Nude and SCID-Beige	[165]
BI-505	ICAM-1	B cell cancer and MM	SCID	[166]
MLN4924	NEDD8-Activating Enzyme	Multiple cancers	SCID	[167]
Selinexor (KPT-330)	XPO1	AML	NSG	[168]
Matrix metalloproteinase inhibitor prinomastat (AG3340)	Matrix metalloproteinase	Pancreatic ductal adenocarcinoma	SCID	[169]

### Identification of cancer biomarkers

PDX models in preclinical cancer research is to aid the identification of cancer-specific biomarkers that can be used for diagnosis, prognosis, and therapeutically targeted. Whole-transcriptome profiling of PDX models to identify both tumor- and stromal-specific biomarkers supports drug efficacy studies and compartment-specific biomarker discovery [77]. PDX models have been used to evaluate possible detective agents for the diagnosis of cancers, such as the fluorescently labeled chimeric anti-CEA antibody in the detection of colon cancer [78]. The prognostic value of stem cell markers in cancers such as hepatocellular carcinoma (HCC) [79] has been evaluated in PDX models. For cancers such as bladder cancer, PDX models are useful both for the discovery of novel molecular targets and predictive biomarkers and for determining the risk of treatment failure [80]. Generation of paired chemonaive and chemoresistant small cell lung cancer (SCLC) PDX models led to the finding that EZH2 promotes chemoresistance by epigenetically silencing SLFN11, and EZH2 inhibition prevents acquisition of chemoresistance and improves chemotherapeutic efficacy in SCLC [81]. NEK2 represents a strong predictor for drug resistance and poor prognosis in cancer, in that targeting NEK2 by NEK2 shRNA overcame drug resistance and induced apoptosis in vitro and in a myeloma PDX model [82]. The long non-coding RNA gene *SAMMSON* can be targeted to sensitize melanoma to MAPK-targeting therapeutics both in vitro and in PDX models [83]. The IGF-1 receptor is universally expressed in various cancers, which can be therapeutically targeted, as exemplified by an orthotopic PDX model of multiple myeloma [84].

### Identification and evaluation of potential drugs

#### Chemicals

Conventional chemotherapy is still the mainstay treatment modality for various cancers, and PDX models are valuable tools for the evaluation of chemical drugs in vivo. PDX models have been used to evaluate dozens of small-molecule compounds, mainly kinase inhibitors, in various cancers. Kinase inhibitors have been tested in PDX models for cholangiocarcinoma [85], chordoma [86], NSCLC [87], gastric cancer [88], etc. VEGF blocker FP3 inhibited gastric cancer through an antiangiogenic mechanism in a PDX model [89]. CXCR4 is critical to T-ALL cell leukemogenicity and required for T-ALL migration, homing, and niche positioning [90]. And targeting CXCR4 with small-molecule antagonists reduces tumor growth in murine T-ALL and T-ALL PDX models [91]. Inhibition of the MDM2–p53 interaction suppressed tumor growth in PDX models for NSCLC [92]. Inhibition of MTH1 selectively causes incorporation of oxidized dNTPs in cancer cells, leading to DNA damage, cytotoxicity, and therapeutic responses in patient-derived mouse xenografts [93]. Gesterone receptor antagonists show antiproliferative and proapoptotic activities in breast cancer PDX models [94]. Luteolin inhibits tumor growth in cMet-overexpressing PDX models of gastric cancer [95]. The compound trabectedin modulates gene and microRNA expression and various signaling pathways in PDX models [96]. PF-06463922, a potent and brain-penetrant ALK/ROS1 inhibitor, displayed superior potency against all known clinically acquired ALK mutations and inhibited regression of EML4-ALK-driven brain metastases and prolonged survival of PDX mice [97].

#### Antibodies

Moreover, PDX models are valuable tools for the tests of novel antibodies before clinical application. Antibody-based therapies have been widely used in the clinical treatment of cancer patients, and PDX models have been used to test the use of antibodies for the treatment of various cancers [98, 99]. Especially, immune checkpoint blockade therapy (ICBT), which blocks PD-1, PD-L1, or CTLA4 with antibodies, has elicited a remarkable clinical response in certain cancer patients. We recently evaluated new PD-1/PD-L1 antibodies in NSCLC PDX models established in humanized NSI mice reconstituted with human HSC or blood cells (unpublished). Nevertheless, intrinsic resistance to immune checkpoint inhibitors remains a daunting challenge [100]. PDX models can be used to evaluate treatments targeting specific resistance mechanisms to sensitize ICBT-resistant tumors. As for other antibodies, NSCLC PDX models with genetic aberrations within EGFR, KRAS, and FGFR1 were used to evaluate the range of responses to Gefitinib, which were shown in vivo to be consistent with the results of clinical trials [66]. In a human bladder cancer PDX model, bladder cancer stem cells (CSCs) actively contribute to therapeutic resistance, which can be abrogated by a PGE2-neutralizing antibody and celecoxib drug-mediated blockade of PGE2 signaling [101].

#### Anti-cancer microorganisms

PDX models are valuable tools for the careful assessment of attenuated microorganisms in cancer treatment. *Salmonella typhimurium* A1-R, a facultative anaerobe that can grow in the oxic viable region of tumors and in necrotic regions, has shown efficacy against osteosarcoma [102], soft-tissue sarcoma [103], and melanoma [104] in orthotopic PDX models. And the oncolytic viruses are also promising for cancer treatment. The attenuated vesicular stomatitis strains, AV1 and AV2, were tested in a xenograft model of ovarian cancer, which effected complete and durable cures in the majority of treated animals when delivered systemically [105]. Oncolytic virus Delta24-RGD [106] and measles virus strains [107] have been tested in PDX models for glioblastoma.

#### Drug combinations

Targeted cancer therapies often lead to resistance, which can be suppressed through combination drug therapies. Combinatory targeting of two or more onco-signaling pathways is a promising strategy for cancer therapy. We recently used B-ALL PDX models to evaluate the anti-B-ALL efficacy of the combination of disulfiram and copper [108]. PDX models are useful for defining the optimal target combinations which avoid therapy resistance, as has been done in the glioblastoma PDX model through single-cell phosphoproteomics [109]. CDK4/6 inhibitors resensitize PDX tumors to HER2-targeted therapies and delay tumor recurrence [110]. Combination treatment with the Aurora kinase A inhibitor MLN8237 and ABT-199 is synergistic in PDX models of MYCN-amplified neuroblastomas [111]. Combined CDK4/6-PI3K inhibition overcomes intrinsic and adaptive resistance leading to tumor regressions in PIK3CA mutant breast cancer PDXs [112]. BRAF (V600E) mutant colon cancers may benefit from a combination therapy consisting of BRAF and EGFR inhibitors; EGFR and BRAF (V600E) inhibitors synergize to induce apoptosis of colorectal cells and to suppress colorectal tumor growth in a xenograft model [113]. Anti-CD47 antibody synergized with rituximab, by promoting phagocytosis, to eliminate lymphoma in both disseminated and localized non-Hodgkin lymphoma (NHL) xenograft models [114].

## High-throughput drug screening and assessment

A major issue in cancer drug development is the low success rate of new agents. Many compounds advance to large phase III studies, which consume considerable resources, but eventually fail because of low efficacy. These poor results arise partly because conventional preclinical models to screen new agents for clinical development have poor predictive value [115]. Furthermore, new drugs are tested in patients without selection and response monitoring through appropriate biomarkers. In this regard, the availability of PDX models with high predictive value is of major interest. The ex vivo cultured PDX tumor cells can be used for the in vitro high-throughput screening of anti-cancer drugs (Fig. 2) [116]. PDX models theoretically can provide unlimited sources of human tumor cells for ex vivo high-throughput drug assessment. A large biobank of breast cancer PDXs, which preserves morphological and molecular characteristics and intra-tumor genomic clonal architecture of the originating tumors, has been generated and used for high-throughput drug assessment in PDX-derived tumor cells in vitro [117]. The Public Repository of Xenografts (PRoXe) is a publicly available repository of well-characterized leukemia and lymphoma PDXs, which can be used to characterize drug efficacy and generate transcriptional, functional, and proteomic biomarkers in both treatment-naive and relapsed/refractory disease, and randomized phase II-like studies with PRoXe are applicable to a range of therapeutic agents, especially those that act through cancer cell-intrinsic mechanisms [118]. PDX models are also useful for assessment of drugs screened from high-throughput computational design. A novel computational design approach yields multivalent pan-RAS inhibitors and PDX models were used to confirm the efficacy of the identified small-molecule compound binding to KRAS^G12D^ [119]. Another computationally designed protein BINDI, binding with BHRF1 of Epstein-Barr virus, suppressed tumor growth and extended survival in a PDX model of EBV-positive human lymphoma [120].

## CAR T cell immunotherapies

Adoptive transfer of chimeric antigen receptor (CAR) T cells has shown great promise in treating cancers, especially in B cell leukemia with CAR T cells targeting CD19. PDX models are frequently used for preclinical studies of chimeric antigen receptor (CAR) T cells [121–123]. Novel designs of CARs have been frequently evaluated in PDX models. The in vivo model with NSG mice was critical to demonstrate that targeting an anti-CD19 CAR to the *TRAC* locus with CRISPR/Cas9 enhances tumor rejection, a strategy averting antigen-stimulated differentiation and exhaustion [124]. The “On-switch” CARs that enable small-molecule control over CAR T cell therapeutic function as to timing, location, and dosage of T cell activity, thereby mitigating toxicity [125]. Loss of HVEM, which disrupts HVEM-BTLA inhibitory interaction, leads to cell-autonomous activation of B cell proliferation and promotes lymphoma development. So, the anti-CD19 CAR T cells producing HVEM were tested and showed improved anti-lymphoma efficacy in the PDX model [126]. The CAR T cell immunotherapies have not generated satisfactory results in almost all types of solid tumors. PDX models for solid tumors will play essential roles in future studies to promote efficacies of CAR T cells against solid tumors.

In summary, PDX models facilitate the discovery and testing of various therapeutic regimens including small-molecule compounds, antibodies, microorganisms, and cytotoxic cells.

## Discussion

PDX models can provide in vivo evidences to support in vitro findings, and data from PDX models may lead to new discoveries or hypotheses which can be further investigated by research in vitro. The use of these xenograft models to study human tumor biology and drug screening is, however, limited by several factors, including the replacement of human stromal components (such as cancer-associated fibroblasts, endothelial cells, immune and inflammatory cells) by murine elements, the lack of a functional immune system, and the lack of interactions between human stromal cells and the immune system. The development of PDX models that account for interactions between tumor, stromal, vascular, and immune cells is essential to produce a tumor microenvironment more representative of the human host. PDX models in humanized xenochimeric mice, or XactMice, engrafted with human HSPCs before tumor engraftment expressed the chemical stimuli necessary to give rise to stromal and immune cells that recreated the original tumor microenvironment observed clinically [127]; nonetheless, better PDX models are still needed to simulate real cancer–stromal interactions in patients. Furthermore, new approaches to optimizing cancer drug development are required to fully achieve the goal of individualized, precision cancer therapy, and improved preclinical models that more closely reflect the genomic complexity of human cancers are needed.

Recent studies using single-cell sequencing suggest that in some PDX models, only a limited number of clones propagate in mice, indicating a selection process [128]. The identification of lymphocytes recognizing tumor-specific mutant neoantigens represents a major step toward the future eradication of heterogeneous cancers. Only recently reported was the identification of neoantigen-specific lymphocytes in the peripheral blood of melanoma patients [129]. However, the routine detection of lymphocytes that target neoantigens is currently limited to T cells isolated directly from cancer patients, which are often not available. This limitation might be overcome using PDX models produced by engrafting an autologous immune system. With genetically humanized immunodeficient mice which can engraft a more integrate human immune system, we will be able to upgrade the translational research on cancers as well as on other diseases including infectious diseases and autoimmune diseases.

## Conclusions

PDX models are increasingly used in translational cancer research. These models are useful for the study of cancer biology, biomarker development, drug screening, and the preclinical evaluation of personalized medicine strategies. This review provides a timely overview of the key roles of PDX models in both basic and preclinical cancer research and a detailed discussion of major hurdles in the field.

## Abbreviations

CTCs: Circulating tumor cells
FGS: Precise fluorescence-guided surgery
HCC: Hepatocellular carcinoma
NSCLC: Non-small cell lung cancer
PDX: Patient-derived xenograft
